# [^18^F]FDG-PET/CT in mechanically ventilated critically ill patients with COVID-19 ARDS and persistent inflammation

**DOI:** 10.1007/s40336-023-00550-y

**Published:** 2023-03-12

**Authors:** Bram van Leer, Johannes H. van Snick, Mark Londema, Maarten W. N. Nijsten, Ömer Kasalak, Riemer H. J. A. Slart, Andor W. J. M. Glaudemans, Janesh Pillay

**Affiliations:** 1 Medical Imaging Center, Department of Nuclear Medicine and Molecular Imaging, University Medical Center Groningen, University of Groningen, Groningen, The Netherlands; 2 Department of Critical Care, University Medical Center Groningen, University of Groningen, TA29, PO box: 30 001, 9700 RB Groningen, The Netherlands; 3 Department of Radiology, University Medical Center Groningen, University of Groningen, Groningen, The Netherlands; 4 Groningen Research Institute for Asthma and COPD (GRIAC), University Medical Center Groningen, University of Groningen, Groningen, The Netherlands; 5 Biomedical Photonic Imaging Group, Faculty of Science and Technology, University of Twente, Enschede, The Netherlands

**Keywords:** ARDS, COVID-19, Critically ill patients, ICU, Persistent inflammation, [^18^F]FDG-PET/CT

## Abstract

**Purpose:**

We report the findings of four critically ill patients who underwent an [^18^F]FDG-PET/CT because of persistent inflammation during the late phase of their COVID-19.

**Methods:**

Four mechanically ventilated patients with COVID-19 were retrospectively discussed in a research group to evaluate the added value of [^18^F]FDG-PET/CT.

**Results:**

Although pulmonary PET/CT findings differed, bilateral lung anomalies could explain the increased CRP and leukocytes in all patients. This underscores the limited ability of the routine laboratory to discriminate inflammation from secondary infections. Based on PET/CT findings, a secondary infection/inflammatory focus was suspected in two patients (pancreatitis and gastritis). Lymphadenopathy was present in patients with a detectable SARS-CoV-2 viral load. Muscle uptake around the hips or shoulders was observed in all patients, possibly due to the process of heterotopic ossification.

**Conclusion:**

This case series illustrates the diagnostic potential of [^18^F]FDG-PET/CT imaging in critically ill patients with persistent COVID-19 for the identification of other causes of inflammation and demonstrates that this technique can be performed safely in mechanically ventilated critically ill patients.

## Introduction

Severe respiratory failure due to SARS-CoV-2 infection that requires admission to the intensive care unit (ICU) is often complicated by acute kidney injury, multiple organ failure, thrombosis, and secondary infections contributing to prolonged length of stay, morbidity, and mortality. Persistent inflammation is a hallmark of most patients with a prolonged ICU stay. This inflammation can be present when viral loads have diminished and the patient has cleared SARS-CoV-2 [1]. Diagnosing the source of inflammation in these patients, either ongoing SARS-CoV-2-induced inflammation or foci of secondary infections and inflammation, is challenging as classical inflammatory parameters, such as CRP and leukocyte count, are elevated and non-specific.

[^18^F]fluorodeoxyglucose ([^18^F]FDG) positron emission tomography/computed tomography (PET/CT) is a well-known imaging technique able to identify sites with increased metabolism for the detection of infections and/or ongoing inflammation, but is hardly used in critically ill patients [2–4]. It may help to identify sources of persistent inflammation after SARS-CoV-2 infection [2, 5–8]. Many small studies already demonstrated that COVID-19 pneumonia can be visualized with [^18^F]FDG-PET/CT, even in asymptomatic patients [8–16]. However, to our knowledge no reports exist evaluating the use of [^18^F]FDG-PET in ICU patients with COVID-19 acute respiratory distress syndrome (ARDS). We have previously demonstrated that the use of PET/CT in critically ill patients is feasible and safe [4]. Here we report the findings of four ICU patients with COVID-19 ARDS who underwent an [^18^F]FDG-PET/CT for the diagnosis of foci of persistent inflammation.

## Methods

Between April 2020 and March 2022, four mechanically ventilated patients with COVID-19 ARDS admitted to the ICU of the University Medical Center Groningen underwent an [^18^F]FDG-PET/CT. These cases were prospectively and retrospectively evaluated (for the purpose of this study) in a research group consisting of senior nuclear medicine physicians (AG and RS), a senior radiologist with expertise in lung fibrosis imaging (OK) and intensivists (JP and MN). Scanning was performed on the Biograph mCT 64, 14 bed positions in 30 min or Biograph Vision Quadra, 1 bed position in 10 min (Siemens Healthineers, Erlangen, Germany). PET/CT imaging was performed approximately 60 min after intravenous [^18^F]FDG administration (± 3 MBq/kg). Low-dose CT was performed for attenuation correction and anatomic mapping with Care kV 100 kV and Quality ref mAs of 30. High-dose CT was performed with 140 kV and 75 mAs. Visual analyses were performed using Syngo.via VB 50 (Siemens Healthineers, Erlangen, Germany). Patient data were retrospectively collected via electronic patient files, including laboratory findings. The study was approved by the local institutional review board and waived the requirement for written informed consent for this study.

## Results

Three males and one female with ages ranging from 26 to 65 underwent an [^18^F]FDG-PET/CT scan. In all four patients, the indication for a PET/CT was persistent elevation of CRP and the leucocyte count, accompanied by a deterioration in respiratory and/or vital functions in the post-acute phase of their COVID-19 ARDS (between day 18 and 37 after intubation). Decision to perform PET/CT imaging was at the discretion of the attending clinician. Three of the four patients were scanned between November 2021 and February 2022 after installation of a new long axial field of view scanner. None of the patients had an obvious source of infection (apart from SARS-CoV-2) at the time of PET/CT procedure and two patients had already tested negative (PCR cycle threshold value > 35) for SARS-CoV-2 at the time of the scan. All patients were mechanically ventilated using lung protective settings with the use of prone positioning according to ARDSnet recommendations [17]. In addition, one patient received rescue therapy with veno-venous extracorporeal life support. One patient passed away during admission due to progressive respiratory failure and accompanying multiple organ failure 4 days after the scan. Patient characteristics are shown in Table 1.

**Table 1:** Patient characteristics

	Patient 1	Patient 2	Patient 3	Patient 4
Age (year)	65	46	27	26
Gender	Male	Male	Male	Female
BMI	24.1	29.0	26.3	26.0
Admission reason	Respiratory failure	Respiratory failure	Respiratory failure	Respiratory failure
Vaccination status	Not vaccinated	Not vaccinated	Not vaccinated	Not vaccinated
Immune compromised	Yes	No	No	No
Died during admission	No	Yes	No	No
Number of ventilation days	24	25	61	36
VFD-28	4	0	0	0
PEEP at time of PET cmH2O	8	14	12	14
FiO2 at time of PET %	35	65	30	40
ARDS severity at time of admission	Moderate	Severe	Moderate	Severe
ARDS severity at time of PET/CT	Mild	Moderate	Mild	Mild
Prone position	Yes	Yes	Yes	Yes
ECLS	No	No	Yes	No
Steroids at admission	20 mg prednisolone daily	10 days of 6 mg dexamethasone	10 days of 6 mg dexamethasone	10 days of 6 mg dexamethasone
Anti-IL-6 at admission	No	Yes	Yes	Yes
COVID-19 status at time of PET	Negative	Positive	Negative	Positive
Leukocyte count at time of PET [10^**9**^/L]	7.4	14.9	15.4	15.0
CRP at time of PET [mg/L]	150	274	200	223
Therapy changes based upon PET	No	No	Yes, additional steroid treatment	No
Other modalities applied during ICU admission	Chest X-ray, abdominal ultrasound	Chest X-ray, pulmonary CTA, bronchoscopy	Ultrasound for deep venous thrombosis, pulmonary CTA, CT brain, abdominal ultrasound, bronchoscopy	Pulmonary CTA, abdominal CT, abdominal ultrasound, bronchoscopy

*BMI* body mass index, *VFD-28* ventilation-free days of 28 days, *PEEP* positive end expiratory pressure, *ECLS* extracorporeal life support

### Pulmonary findings

**Fig. 1:**
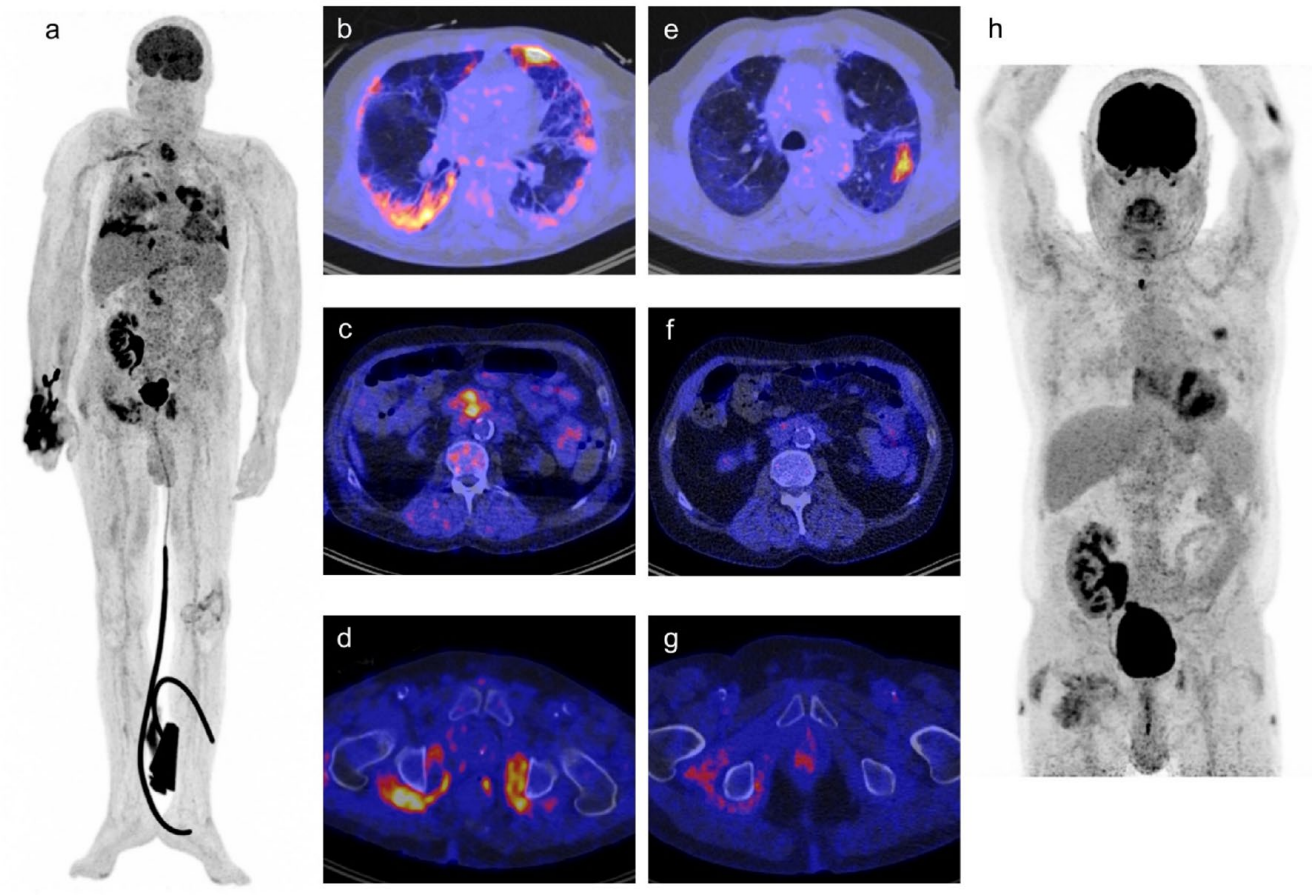
[^18^F]FDG-PET/CT scan of patient 1. This concerns a 65-year-old man who was admitted to the ICU 2 weeks after experiencing symptoms of COVID-19 at the start of the pandemic. An [^18^F]FDG-PET/CT scan was performed 18 days after intubation due to ongoing high CRP levels and negative PCR results for SARS-CoV-2, with no other identified focus of infection. Although not at the time, retrospectively a pancreatitis seems likely due to absence of uptake at the follow-up [^18^F]FDG-PET/CT and high amylase at the time of ICU admission. The [^18^F]FDG-PET/CT did not lead to therapy changes and the patient recovered slowly. [^18^F]FDG-PET/CT revealed high uptake in the muscles in the hip region, which was finally diagnosed as heterotopic ossification. Initial scan: **a** Maximum intensity projection. Physiological uptake in the right-sided donor kidney, liver, bladder and brain. Percutaneous tracer injection in the right hand. Uptake below the cricoid due to a tracheostomy. Some muscle uptake in the shoulders. **B** Transaxial pulmonary fusion PET/CT image with clear [^18^F]FDG uptake in the peripheral areas of the lung. CT in lung setting. **C** Transaxial abdominal fusion PET/CT with [^18^F]FDG uptake in the pancreatic head. CT in soft tissue setting. **D** Transaxial fusion PET/CT image of the hip region with [^18^F]FDG uptake in the dorsal muscles. CT in bone setting. Follow-up scan: **E** Transaxial pulmonary fusion PET/CT with only one active lesion left. CT in lung setting. **F** Transaxial abdominal fusion PET/CT, [^18^F]FDG uptake in the pancreas disappeared. CT in soft tissue setting. **G** Transaxial fusion PET/CT of the hip with decreased [^18^F]FDG uptake in the dorsal muscles. On CT, some linear calcifications are visible in the right dorsal hip muscles. CT in bone setting. **H** Maximum intensity projection of follow-up scan. Physiological uptake in the donor kidney, liver, and spleen. Tracheostomy uptake decreased. *SUV-bw threshold of 5.00; α* = *50%*

**Fig. 2:**
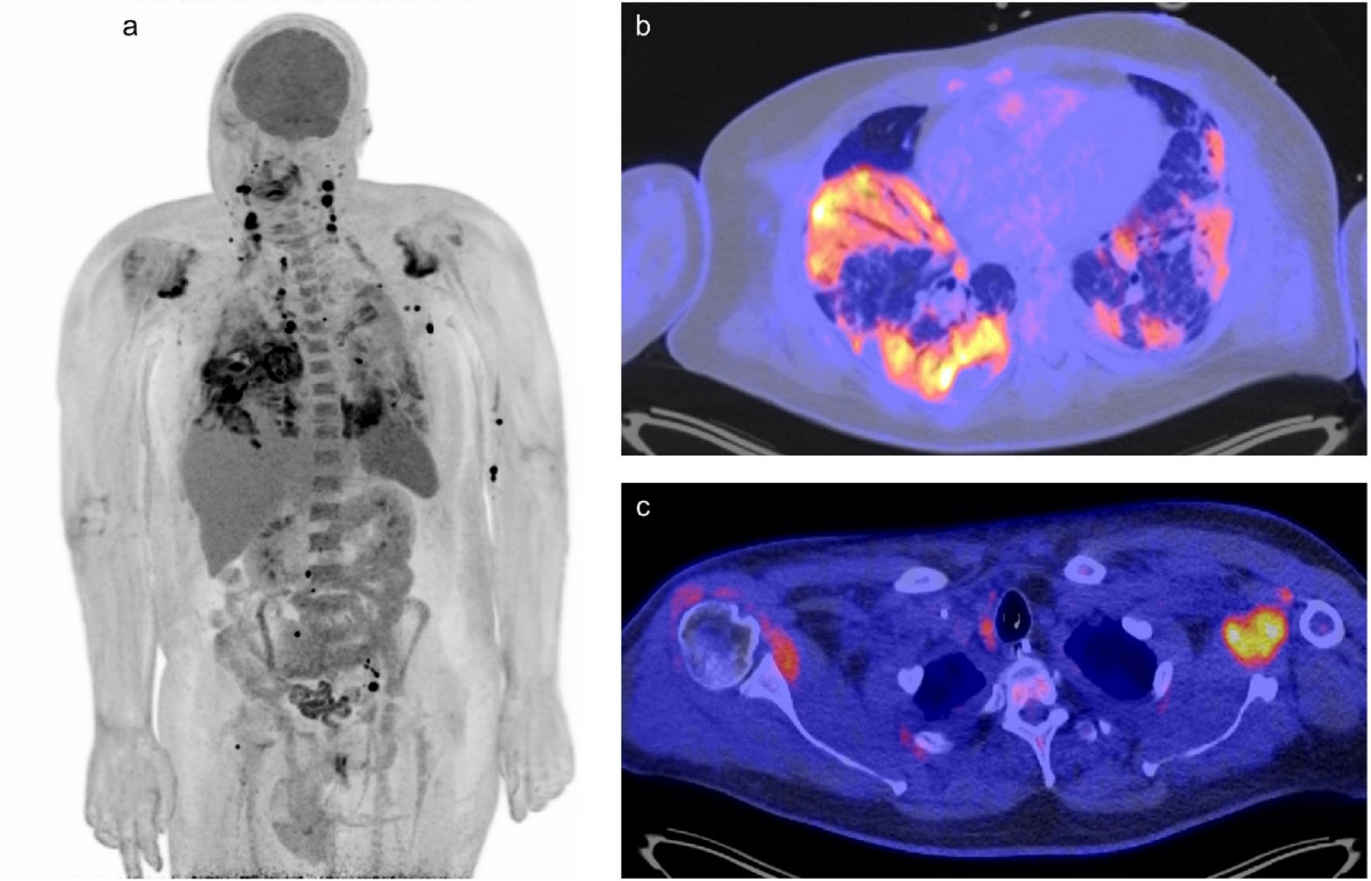
[^18^F]FDG-PET/CT of patient 2. A 46-year-old male was admitted to the ICU 1 week after developing symptoms of COVID-19. Due to ongoing inflammation without focus and deteriorating multiorgan failure, an [^18^F]FDG-PET/CT was performed to evaluate the presence of a secondary infection. No infection focus was found and the patient stayed SARS-CoV-2 positive till his dead. **a** Maximum intensity projection. Physiological uptake in the liver and intestines. Atypical low intensity in the kidneys due to kidney failure. Reduced uptake in the brain due to sedation. Reactive uptake in the spleen and bone marrow. The lymphadenopathy can be seen very clearly. **b** Transaxial pulmonary fusion PET/CT image with [^18^F]FDG uptake in the peripheral and central areas of the lung. CT in lung setting. **c** Transaxial fusion PET/CT image of the shoulder regions with [^18^F]FDG uptake in the anterior medial muscles of the shoulders (left more than right). CT in soft tissue setting. *SUV-bw threshold of 5.00; α* = *50%*

**Fig. 3:**
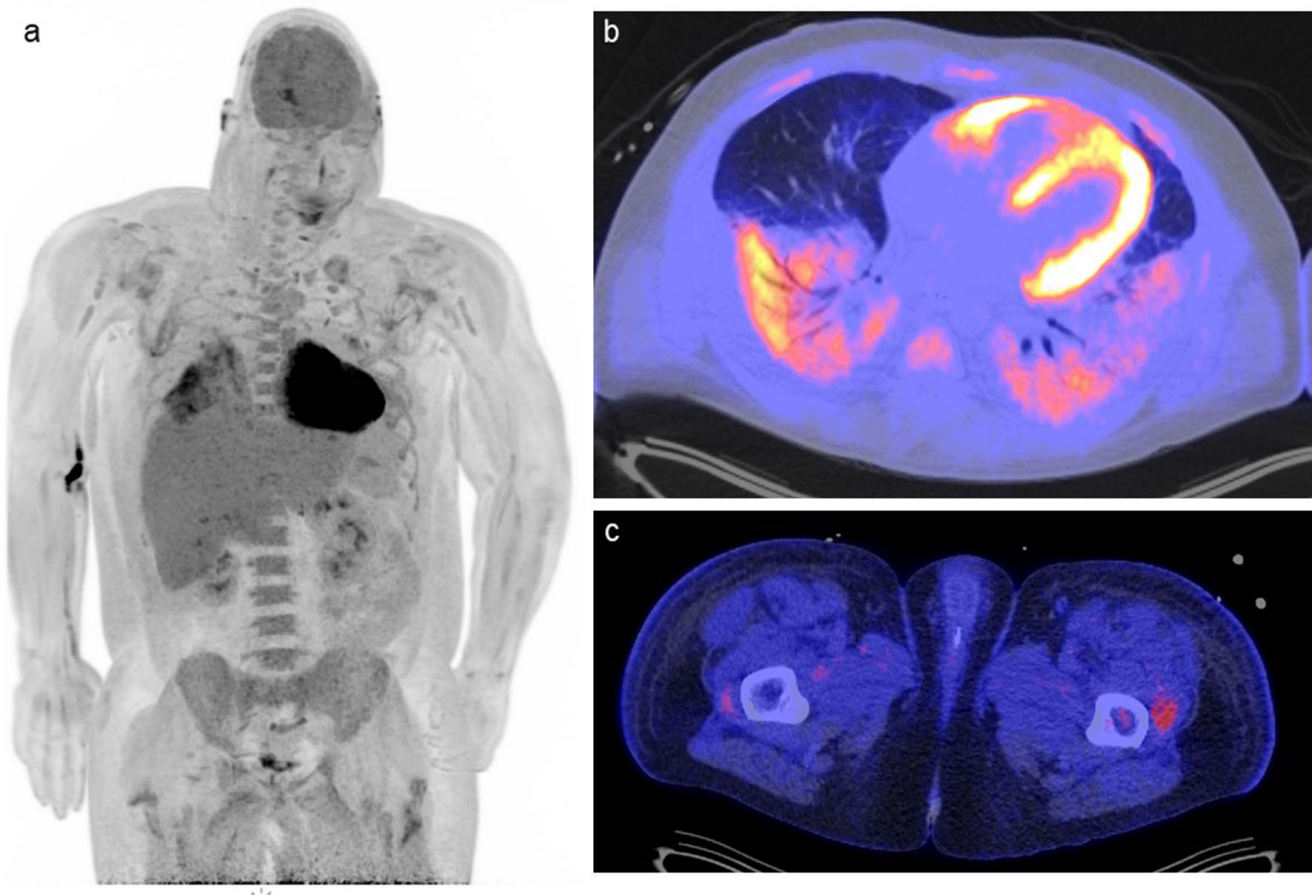
[^18^F]FDG-PET/CT of patient 3. A 27-year-old male was intubated 10 days after the onset of symptoms of COVID-19. Mechanical ventilation was partially combined with extracorporeal membrane oxygenation due to worsening of the patient's respiratory condition. When the patient’s condition deteriorated again, a second treatment with extracorporeal membrane oxygenation was not possible due to inflammation. An [^18^F]FDG-PET/CT was performed to identify a potential focus of infection. However, while the patient tested negative for SARS-CoV-2, no clear focus was identified on the PET/CT, transesophageal echo, or bronchoalveolar lavage. The patient was therefore treated with high-dose steroids, leading to a slow recovery. The patient was ventilated for over 2 months in total. **a** Maximum intensity projection. Physiological uptake in the liver. Atypical low intensity in the kidneys due to kidney failure. Reduced uptake in the brain due to sedation. There is no suppression of the heart, most likely due to short and insufficient fasting time. **b** Transaxial pulmonary fused PET/CT image with peripheral inferior atelectasis and consolidations on CT with [^18^F]FDG uptake. CT in lung setting **c** Transaxial fusion PET/CT of the hip with some slightly elevated uptake in the lateral muscle of the left hip. CT in soft tissue setting. *SUV-bw threshold of 5.00 α* = *50%*

**Fig. 4:**
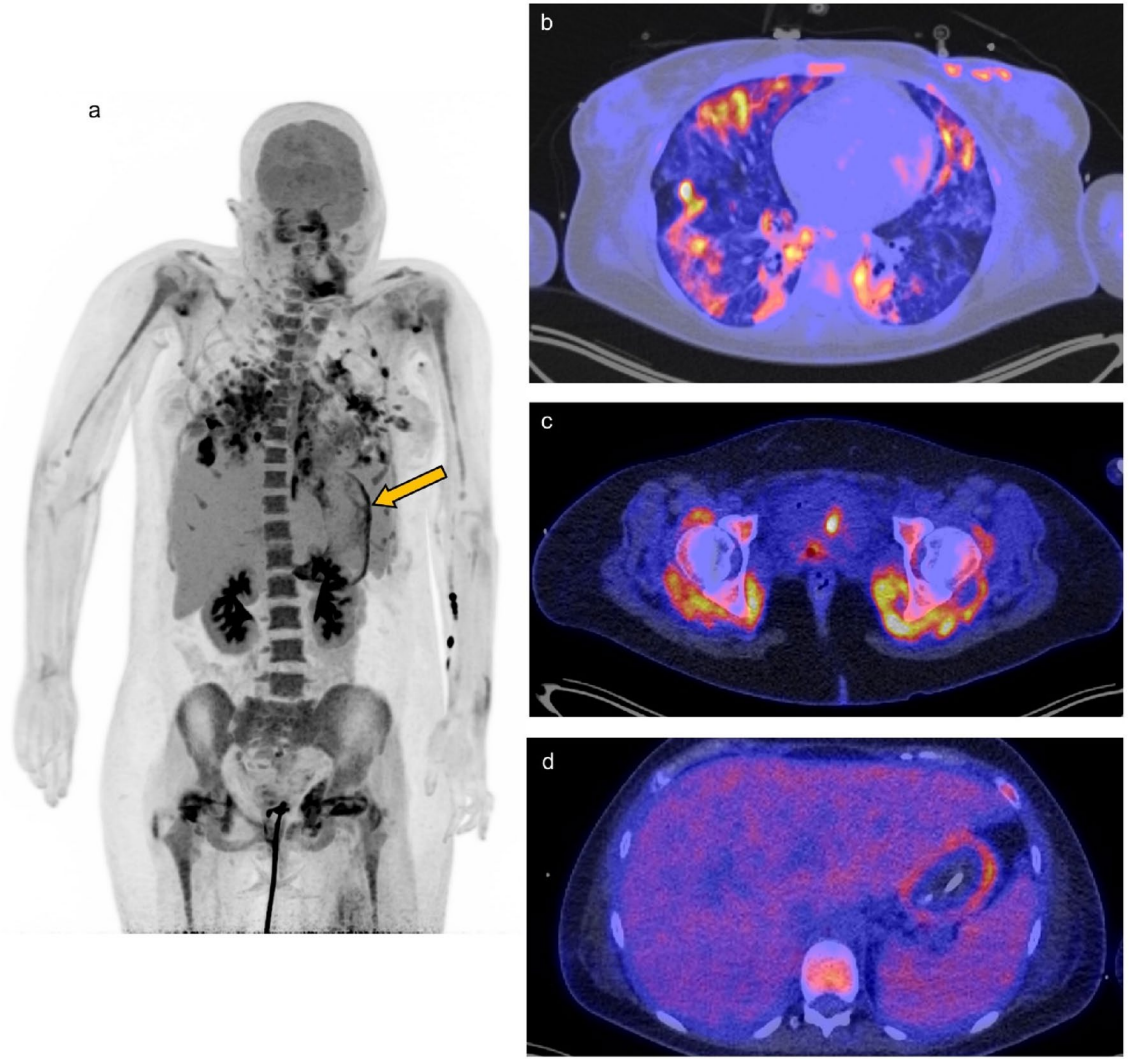
[^18^F]FDG-PET/CT of patient 4. The last patient is a 26-year-old pregnant female who was admitted because of mild respiratory failure due to COVID-19 and premature contractions wherefore a C-section was performed. Two days after C-section, the patient became severe respiratory insufficient and was mechanically ventilated. Due to a second episode of respiratory decline and ongoing inflammation, an [^18^F]FDG-PET/CT was performed. A gastritis was found as a possible secondary infection focus. The patient was still SARS-CoV-2 positive at the time of [^18^F]FDG-PET/CT. **a** Maximum intensity projection. Physiological uptake in the kidneys and liver. Reduced uptake in the brain due to sedation. Reactive bone marrow. The yellow arrow points toward the uptake in the gastric wall. **b** Transaxial fusion PET/CT image of the lungs with ground-glass opacities and some consolidations on CT, characteristic for COVID-19 ARDS. Heterogenous uptake of [^18^F]FDG. CT in lung setting. **c** Transaxial fusion PET/CT image of the hips with high uptake in the posterior lateral and medial muscle of the hip at both sides. CT in soft tissue setting. **d** Transaxial fusion PET/CT image of the abdomen with clear [^18^F]FDG uptake in the gastric wall. *SUV-bw threshold of 5.00 α* = *50%*

The pulmonary [^18^F]FDG uptake profile varied strongly between the patients. Patient 1 showed peripheral uptake in all five lung lobes in a total of ten active lesions, encompassing less than 25% of the total lung area. A low-dose CT (LDCT) in the lung window setting showed ground-glass opacities and consolidations, which were more widespread than the [^18^F]FDG-affected areas (Fig. 1). In patient 2, only three of the five lobes were involved, with a total active lung area of 25–50%, localized peripherally as well as centrally. LDCT showed large consolidations as well as ground-glass opacities and pleural effusion, mostly corresponding with the [^18^F]FDG activity (Fig. 2). For patient 3, [^18^F]FDG uptake was limited to the inferior lobes with matching LDCT abnormalities (consolidations, atelectasis and tree-in-bud pattern), suggesting metabolically active processes inside these affected areas (Fig. 3). In patient 4, high-resolution CT (HRCT) scanning showed a large area of ground-glass opacities and some atelectasis and consolidations, fitting typical imaging of SARS-CoV-2-infected lungs. [^18^F]FDG activity lesions were diffusely spread throughout the lung and not all CT abnormalities showed [^18^F]FDG activity (Fig. 4). All PET procedurals and findings are shown in Table 2.

**Table 2:** PET procedurals and findings per patient

	Patient 1	Patient 2	Patient 3	Patient 4
General				
Time of PET in days after intubation	18	21	37	22
PET scanner	Biograph mCT 64	Biograph Vision Quadra	Biograph Vision Quadra	Biograph Vision Quadra
Doses	210 MBq	290 MBq	330 MBq	240 MBq
Scan quality	Moderate	Good	Good	Good
Preparation diet	Fasting	Ketocal 4:1	Fasting	Ketocal 4:1
Glucose level at time of PET mmol/l	6.1	7.6	6.9	5.7
Insulin administration rate at time of PET IU/h	0	0	0	0
RASS	0	− 5	− 2	− 5
Follow-up scan	Yes	No	No	No
Lung				
Total lung activity	< 25%	25–50%	< 25%	25–50%
Involved lung lobes	5/5	3/5	2/5	4/5
Total number of active lesions	10	6	2	9
Location lesions	Peripheral	Peripheral, central	Peripheral	Peripheral, central
LDCT findings	Ground-glass opacities, consolidations	Ground-glass opacities, consolidations, pleural emphysema	Atelectasis, pleura emphysema, three-in-bud pattern, consolidations, ground-glass opacities	Ground-glass opacities, atelectasis, consolidations
Heart and vessels				
Heart suppression	Partial	Almost full	No suppression	Almost full
Vasculitis	No	No	No	No
Lymph nodes				
Lymph node location	Mediastinal	Supraclavicular, mediastinal, axial, para-aortic, para-iliac	NA	Mediastinal, para-costal
Muscle and joints				
Paravertebral				
Shoulder		Both sides	Both sides	Both sides
Hip	Both sides		Both sides	Both sides
Proximal leg			Both sides	
Culprit				
Location	Pancreas head	None	None	Gastric wall

*RASS* Richmond Agitation Sedation Scale

### Extrapulmonary findings

Intense [^18^F]FDG uptake around the pancreatic head with unclear etiology was noticed in patient 1 which was initially not differentiated. Retrospectively, pancreatitis could be an obvious cause of this uptake, since at the follow-up PET scan no uptake could be seen anymore, and at time of ICU admission amylase was increased (748 U/L, normal value: < 107 U/L). Due to normalized amylase (normally occurring after one week of increase) and no abdominal pain (clonidine 0.048 mg/h and morphine 1.5 mg/h) at the time of PET/CT, there was clinically no suspicion of pancreatitis. Gastritis was diagnosed in patient 4 due to increased [^18^F]FDG uptake in the gastric wall. Gastric bleeding at time of admission and gastroscopy showing vulnerable mucosa strengthened the likelihood of this diagnosis.

Both SARS-CoV-2-positive patients at time of the scan showed multiple [^18^F]FDG-avid lymph nodes at different anatomical areas, whereas in one negative patient only some small mediastinal lymph nodes were noticed. The other negative patient showed no metabolically active lymph nodes.

Striking muscle uptake of [^18^F]FDG was observed around the hip and/or shoulders in all four patients (Figs. 1D, 2C, 3C, 4C). At the follow-up [^18^F]FDG-PET/CT scan in patient 1, performed almost 2 months after the initial scan, calcification of the muscles was seen around the ischium, corresponding to the previous [^18^F]FDG uptake, which was still present at follow-up, although with lower intensity. No information of impaired hip joint movement or pain could be found in the patient files. Muscle weakness was noted. Follow-up (PET/)CT scans in the other patients were not performed.

In two out of the four patients, [^18^F]FDG activity was visible at the skin of the head, most likely due to decubitus caused by prone positioning.

In patient 3, high cardiac uptake was noticed which is most likely due to inadequate preparation for cardiac imaging (only a short fasting time of 12 h and uncertainly if fasting rules were sufficiently followed (Fig. 3A and B)).

### Follow-up

Patient 1 recovered shortly after the PET–CT and was weaned from the ventilator 11 days later. No specific treatment was given for the possible pancreatitis. Patient 2 was still suffering from a SARS-CoV-2 infection and eventually died due to progressive respiratory failure accompanied by extrapulmonary organ failure. Patient 3 was treated with high-dose corticosteroids after an infectious source was considered sufficiently excluded by PET/CT, negative blood cultures, a negative bronchoalveolar lavage, and trans-esophageal echography of the heart. After corticosteroids were initiated, patient 3 slowly recovered. Patient 4 was referred to another hospital the day after the PET/CT and she was weaned from mechanical ventilation 13 days later.

## Discussion

This is the first case series describing [^18^F]FDG-PET/CT findings in mechanically ventilated COVID-19 ARDS patients with persistent inflammation admitted to the ICU. The pulmonary abnormalities observed with [^18^F]FDG-PET/CT differed markedly between the four patients, which suggests different etiologies. For instance, the clinical profile of patient 4 fitted a still active COVID-19 ARDS with a high viral load and classical characteristics on HRCT. This was combined with diffuse [^18^F]FDG activity throughout the entire lung. In contrast, the findings in patient 3—who had become COVID negative—showed mainly atelectasis and some consolidations in the inferior lobes. This may primarily result from the inflammatory sequelae and damaged lung tissue due to SARS-CoV-2 and mechanical ventilation. Noteworthy, atelectasis by itself does not show [^18^F]FDG activity [18]. As no positive microbial cultures were obtained from the bronchoalveolar lavage, it is tempting to speculate that this increased uptake was caused by a persistent inflammatory state such as excessive fibroproliferation, which is potentially responsive to corticosteroids [19]. This theory is supported by the findings that patients with post-COVID-19 lung disease treated with high dose of steroids had a significant lower [^18^F]FDG uptake than those who did not receive such a treatment [12]. The other patient with a detectable SARS-CoV-2 viral load, patient 2, demonstrated a more intense uptake throughout the whole lung than patient 4, suggesting a more severe ARDS [20]. Interestingly, patient 1 showed only peripheral activity whereas patient 2 and 4 showed central localizations as well, although peripheral localization of CT abnormalities is more common in COVID-19 patients [21]. In all four of our patients, a more widespread and intense uptake was seen in comparison to cases in other studies [9, 10, 12–14]. Furthermore, CT abnormalities seem to match with [^18^F]FDG uptake in all those examples, whereas in our case series there is significant mismatch between CT abnormalities and [^18^F]FDG uptake.

Importantly, this pulmonary inflammation could account for the ongoing systemic inflammation in all patients. This underscores the limited ability of the routine laboratory parameters CRP and leukocyte count to discriminate inflammation from secondary infections. This is apparent in the two patients in which possible additional inflammatory foci were found on PET/CT, respectively, a gastritis and pancreatitis. Although concurrent SARS-CoV-2 and pancreatitis have been described, a causal link has not been found [22, 23]. Since all four patients showed high systemic inflammatory parameters on routine laboratory assessment, [^18^F]FDG-PET/CT is of potential added value for the diagnosis of secondary infections [4].

CT abnormalities were more widespread than [^18^F]FDG pulmonary uptake in all patients, underscoring that active inflammatory and steady-state (non-inflammatory) abnormalities cannot be differentiated on a CT scan. Furthermore, lymphadenopathy was present in patients with ongoing SARS-CoV-2 infection, which has been described previously on CT scans performed in critically ill patients and on PET/CT in non-critically ill COVID-19 patients and as a vaccine response [9, 24–26].

All four patients showed muscle uptake around the hips and/or shoulders. This is especially interesting since two of those patients were deeply sedated, therefore active muscle use, explaining physiological [^18^F]FDG uptake, would have been unlikely. The first patient showed heterotopic ossification (HO) at a follow-up PET/CT scan. This was not confirmed by histopathology, and the diagnosis was settled based on typical CT findings. HO in COVID-19 has recently been described in small studies and case reports [27–31]. The largest study by Stoira et al. looked into 52 patients who were mechanically ventilated due to COVID-19 ARDS and showed an incidence of 19% of HO [30]. This was associated with longer duration of mechanical ventilation and hospital stay, as well as creatine kinase levels. Localization of HO occurs most frequently around the hip [30, 32]. Intriguingly, HO due to COVID-19 is only described in critically ill patients [29]. This suggests that ICU-specific supportive care and treatment in addition to mechanical ventilation, such as immobilization, prone positioning and neuromuscular blockage, may contribute to HO [30, 32, 33]. HO is more common in COVID-19 ARDS patients than in non-COVID-19 ARDS patients [30]. In theory, this could be caused by increased duration of mechanical ventilation and sedation [34, 35]. In addition, the influence of systemic inflammation on HO has been described [29, 30, 32, 36]. Our findings suggest that [^18^F]FDG-PET/CT could potentially image the development of HO in critically ill patients during admission at an early stage before calcifications become manifest at CT.

In our institution, in-house transportation of critically ill patients to imaging facilities is a routine procedure that is carefully planned to ensure the safety of the patient. During the transport, the patient is accompanied by at least an ICU nurse and doctor. Additionally, extra time is scheduled at the imaging department to accommodate transfer of the patient onto the scanner. PET/CT imaging can be performed safely in mechanically ventilated patients as was shown in this and earlier performed studies [3, 4, 37, 38]. An increase in the use of PET/CT from November 2021 onward, was due to the installation of a long axial field of view (LAFOV) scanner. LAFOV enables shorter scanning time (3 min in total for the whole body) with a reduced radioactive dose and/or superior scan quality [39]. In particular, the considerably shorter scanning time greatly facilitates the implementation of PET/CT in critically ill patients, as the logistical and safety differences between routine CT scanning and PET/CT are greatly reduced. Furthermore, the new generation digital PET/CT scanners are of a higher quality, possibly increasing the diagnostic value even further by the possibility to show even low-grade inflammation or infection.

Previous case series and retrospective studies have assessed local pulmonary inflammation in COVID-19 [8, 10–16]. However, these studies included mostly asymptomatic patients, in which lung [^18^F]FDG uptake was found as a coincidental finding or in post-COVID-19 patients with persistent respiratory complaints. The only prospectively study did not include critically ill patients [9]. To our knowledge, this is the first case series including mechanically ventilated patients due to critical COVID-19. Furthermore, this study also evaluated extrapulmonary findings, which adds to our understanding of the systemic inflammatory profile and ongoing critical illness in these patients. Moreover, [^18^F]FDG PET/CT data in critically ill patients are rare; therefore, this study increases the limited knowledge of [^18^F]FDG PET/CT findings in critical illness.

Although larger series are needed, and this single center, case series does not provide generalizable conclusions, it illustrates the potential of [^18^F]FDG-PET/CT imaging in ICU patients with (persistent) COVID-19 ARDS.

## Data Availability

Fully anonymized data is available on reasonable request.

## Abbreviations

ICU: Intensive care unit
[^18^F]FDG: [^18^F]fluorodeoxyglucose
PET/CT: Positron emission tomography/computed tomography
ARDS: Acute respiratory distress syndrome
LDCT: Low-dose CT
HRCT: High-resolution CT
HO: Heterotopic ossification
PCR: Polymerase chain reaction
RASS: Richmond Agitation Sedation Scale
